# Age-Related Differences in Associative Learning of Landmarks and Heading Directions in a Virtual Navigation Task

**DOI:** 10.3389/fnagi.2016.00122

**Published:** 2016-05-27

**Authors:** Jimmy Y. Zhong, Scott D. Moffat

**Affiliations:** School of Psychology, Georgia Institute of TechnologyAtlanta, GA, USA

**Keywords:** age-related differences, spatial navigation, route learning, landmark recognition, associative learning, associative memory deficit

## Abstract

Previous studies have showed that spatial memory declines with age but have not clarified the relevance of different landmark cues for specifying heading directions among different age groups. This study examined differences between younger, middle-aged and older adults in route learning and memory tasks after they navigated a virtual maze that contained: (a) critical landmarks that were located at decision points (i.e., intersections) and (b) non-critical landmarks that were located at non-decision points (i.e., the sides of the route). Participants were given a recognition memory test for critical and non-critical landmarks and also given a landmark-direction associative learning task. Compared to younger adults, older adults committed more navigation errors during route learning and were poorer at associating the correct heading directions with both critical and non-critical landmarks. Notably, older adults exhibited a landmark-direction associative memory deficit at decision points; this was the first finding to show that an associative memory deficit exist among older adults in a navigational context for landmarks that are pertinent for reaching a goal, and suggest that older adults may expend more cognitive resources on the encoding of landmark/object features than on the binding of landmark and directional information. This study is also the first to show that older adults did not have a tendency to process non-critical landmarks, which were regarded as distractors/irrelevant cues for specifying the directions to reach the goal, to an equivalent or larger extent than younger adults. We explain this finding in view of the low number of non-critical cues in our virtual maze (relative to a real-world urban environment) that might not have evoked older adults’ usual tendency toward processing or encoding distractors. We explain the age differences in navigational and cognitive performance with regards to functional and structural changes in the hippocampus and parahippocampus, and recommend further investigations into the functional connectivity between the prefrontal cortex and hippocampus for a better understanding of the landmark-direction associative learning among the elderly. Finally, it is hoped that the current behavioral findings will facilitate efforts to identify the neural markers of Alzheimer’s disease, a disease that commonly involves navigational deficits.

## Introduction

Age-related decline in spatial navigation skills, which refers to the ability to learn and navigate in large-scale spaces, has been documented by many studies in both real-world (e.g., Kirasic, 1991; Wilkniss et al., 1997) and virtual environments (VE; e.g., Moffat et al., 2001; Driscoll et al., 2003; Head and Isom, 2010; Harris and Wolbers, 2012, 2014). Behavioral assessments of age-related deficits in navigational ability generally showed that non-demented elderly adults are less proficient than younger adults at learning novel routes (Wilkniss et al., 1997; Moffat et al., 2001), forming and utilizing an allocentric cognitive map for navigating three-dimensional environments (Moffat and Resnick, 2002; Iaria et al., 2009; Wiener et al., 2013), path integration (Mahmood et al., 2009; Adamo et al., 2012; Harris and Wolbers, 2012) and associating landmarks to specific locations or places (Newman and Kaszniak, 2000; Head and Isom, 2010).

Despite these developments, there has been relatively little research examining age-related differences in the associative learning of landmarks and heading directions during route navigation. Utilizing a virtual maze, Head and Isom (2010) showed that younger adults outperformed older adults on a test of landmark-direction knowledge that required the recall of heading directions leading to the finishing point in the maze. The authors suggested that an associative memory deficit (Naveh-Benjamin, 2000; Old and Naveh-Benjamin, 2008) explained older adults’ poorer binding of landmark and direction information. However, the specific details of this potential age-related deficit in associative memory require further investigation as memory for direction-relevant landmarks (i.e., landmarks located at intersections) and direction-irrelevant landmarks (i.e., landmarks located at the sides of one-way passageways) was not assessed separately. Thus, the extent to which younger and older adults differ in the representations of landmark-direction associations for landmarks located at different navigation sites remains unknown.

The investigation of landmarks with regards to differences in their navigational or directional relevance is important because studies have shown that not all landmarks in the environment are perceived and processed in the same fashion. Landmarks that are situated at turns and intersections on a map have been shown to be described more often by people when giving directions of travel to different places (Ward et al., 1986; Blades and Medlicott, 1992) and rated as more important for wayfinding (Lipman, 1991; Daniel and Denis, 1998) than landmarks that are located at the periphery of a route. Assessments of landmark knowledge have also shown that landmarks that are adjacent to correct turns (i.e., turns which lead to the goal point) are better recalled than landmarks that are adjacent to incorrect turns (i.e., turns leading into dead ends) and located by the sides of straight pathways (Cohen and Schuepfer, 1980; Jansen-Osmann, 2002; Jansen-Osmann and Wiedenbauer, 2004; Jansen et al., 2010). Furthermore, in the learning of a route in a virtual maze, Janzen (2006) showed that landmarks/objects located at intersections and turning points (i.e., decision points) were recognized faster than landmarks located at the sides of straight route segments (i.e., non-decision points), and suggested that the differences in recognition time could be attributed to differences in the mental representations associated with the two types of landmarks for navigation purposes.

Previous studies have not examined age group differences in the use of different types of landmark cues for specifying heading directions. Therefore, in this study, we examined and compared navigational and cognitive task performance between younger, middle-aged and older adults in a virtual maze that contained two types of landmark cues: (i) *critical* cues located at decision points (i.e., intersections) where one has to decide on the proper direction to take from several directional options; and (ii) *non-critical* cues located at non-decision points (i.e., along one-way passageways) where deliberate decisions about left- and right-turning directions are not required. In contrast to the critical landmarks which were directly pertinent for learning the right turns to make in order to reach the goal, we regard non-critical landmarks as navigationally irrelevant or even distracting cues that did not facilitate directional decision-making toward the goal. As numerous studies have demonstrated older adults to be prone toward the processing (e.g., Connelly et al., 1991; Duchek et al., 1998; May, 1999; McGinnis, 2012) and preservation of distracting lexical and semantic information compared to younger adults (e.g., Bell et al., 2008; Campbell et al., 2010; Amer and Hasher, 2014), we were further interested to investigate whether older adults would exhibit the same distractor processing tendency in a navigational context, leading to the prediction that older adults would encode/recognize more non-critical landmarks than younger adults. In addition, we included middle-aged adults for our age-group comparisons as Jansen et al. (2010) have shown that they may differ from younger and older adults in terms of route learning and cognitive map formation.

## Materials and Methods

### Participants

A total of 114 participants (58 younger, 29 middle-aged and 27 older adults) participated in this study. The young adults (aged 18–38) were recruited from the psychology research volunteer pool at Wayne State University and from the community. The middle-aged (aged 51–64) and older adults (aged 65–90) were recruited using newspaper advertisements and notices distributed at older adult community centers and events. Based on self-reports of medical history following screening, all participants were in generally good health (i.e., no current history of coronary heart disease, cancer and dementia) and free of any medications that could potentially influence their cognitive performance. None of them suffered from any existing psychiatric or neurological disorders. All of them scored from 27 to 30 on the Mini-Mental State Examination (MMSE) (Folstein et al., 1975), a range that has been shown to offer high classification sensitivity and specificity for ruling out dementia (O’Bryant et al., 2008). With the exception of age and computer gaming experience (see below), older and younger participants were well-matched on demographic characteristics and mental status. Informed consent for this study was approved by the institutional review board at Wayne State University. **Table 1** provides a summary of the demographic information for the sample.

**Table 1:** Demographic characteristics of participants.

	Younger	Middle aged	Older
Age range	18 – 38	51 – 64	65 – 90
Age^a^	20.33 (3.77)	58.79 (4.33)	71.37 (5.45)
% Females	51.7%	44.8%	63.0%
Education^a^	12.90 (1.00)	14.45 (2.17)	14.39 (2.84)
MMSE	29.29 (0.90)	29.41 (0.91)	28.93 (1.11)
CEQ^a^	14.17 (2.85)	11.38 (3.14)	9.15 (3.57)

*Data presented in Mean (±SD). Education: education of participant in years. MMSE, Mini-Mental State Examination. CEQ, Computer Experience Questionnaire. ^a^Variables on which a significant age group difference was present (p < 0.05).*

### Procedures

#### Assessment of Level of Computer Experience

As the elderly participants may have less experience using computers than the younger adults, all participants completed a computer experience questionnaire (CEQ) prior to being tested in the VE. This questionnaire contains three items that asked participants to rate the amount of experience they had using a computer, playing computer games and playing computer games that involve VEs. The scores from each of these items (each rated from 0 to 7) were summed to give a composite computer experience rating for each participant (maximum rating = 21) (see **Table 1**).

#### Pre-Test Training

Before being formally tested, the participants underwent navigation training in a VE. This was done during an initial phase of experimenter instruction, followed by a phase of free exploration of the VE using the joystick. After demonstrating their ability to guide themselves to targets designated by the experimenter, the participants were administered a joystick control speed test. On this test, they navigated a long winding corridor as quickly and accurately as possible until they reached a trophy at the finishing point. The criterion for demonstrating competency in the use of the joystick was completing the task within 120 s. Following the joystick control speed test, the participants conducted navigation in a practice maze over three trials. This maze was similar to the ensuing test maze except that it was of a simpler design.

#### Virtual Maze Learning Task

**FIGURE 1:**
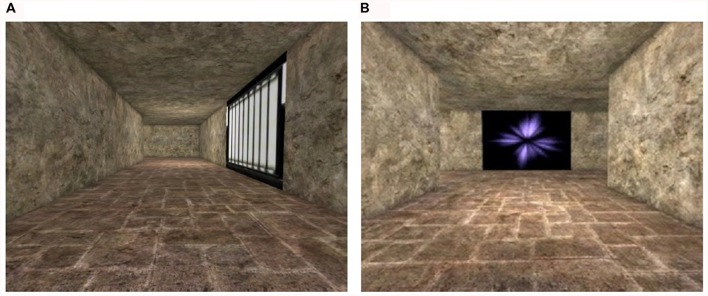
**First-person views of a one-way alley (i.e., non-decision point) (A) and an intersection (i.e., decision point) (B) within the virtual maze.** Landmarks in the form of wall textures with varied colors and patterns were found at each intersection and along certain alleys. Participants were instructed to reach the goal point as quickly and as accurately as possible over five trials.

**FIGURE 2:**
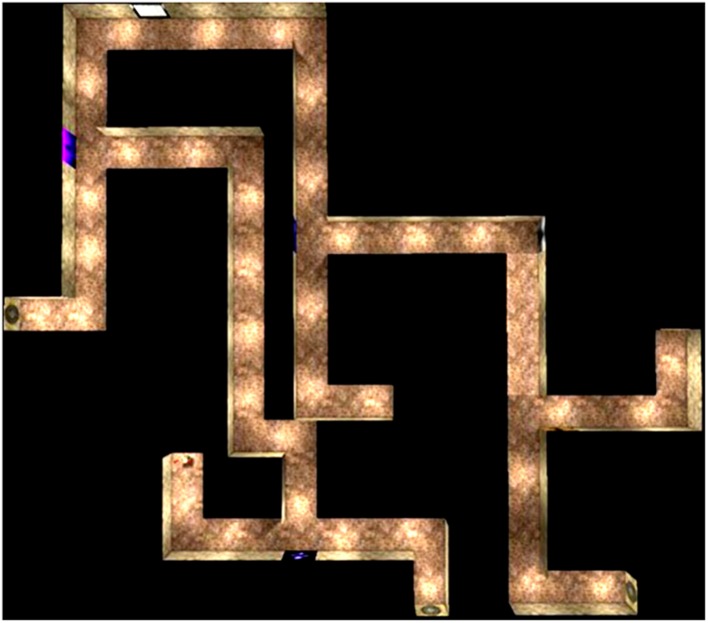
**Overhead view of the virtual maze showing the start and ending points.** There were four intersections or decision points. While making the correct turn at each intersection led one on the right path to the goal point, making an erroneous turn at each intersection led to a dead-end.

The maze learning task was designed using a modified version of Unreal Tournament 2003 and the software package Unreal Editor 3.0 (Epic Games, Inc., Cary, NC, USA). The task was presented on a 21-inch flat panel LCD monitor situated approximately 25 inches in front of the participants. The maze was presented from a first-person perspective and comprised of textured alleys and intersections (see **Figure 1**). There were four intersections at which the participants had to decide about their turning directions into the adjoining alleys. Some of these alleys led to the goal point (marked by the presence of a trophy) while others led to dead ends (for an overhead view, see **Figure 2**). Four intersections were created from pilot testing and showed that they were sufficiently challenging for the older participants. To facilitate maze learning, landmarks in the form of variegated wall textures were positioned at the intersections and along certain alleys. The participants traversed the maze on five consecutive trials after being instructed to locate the goal point as quickly and as accurately as possible and to try to remember the route to the goal over consecutive trials. They navigated the maze using a Logitech WingMan joystick. Aside from being instructed to learn the route to the goal point, they were not given any instruction about the follow-up cognitive tests to be performed. The absence of such instruction allowed for incidental learning of the landmarks for all participants. This ensured that any age effect from the follow-up cognitive tasks would not be overly affected by pre-existing age differences in the cognitive processing of environmental features that could be enlarged through specific instructions of intentional learning (see Old and Naveh-Benjamin, 2008).

The scoring of the maze learning task was automatically done by a program that was specifically developed for recording each participant’s coordinate position and heading bearing in degrees every 20 ms. Based on this record, an analysis of navigation errors was conducted. A navigation error was scored as any deviation from the correct route into an alley that led to a dead end or a journey down a correct route in the wrong direction. An error was noted as soon as the participant crossed the threshold of an intersection into a dead-end alley (i.e., they did not have to reach the end of the alley) and every time they crossed the threshold of an intersection on the correct route but traveling in the wrong direction.

#### Post-Navigation Cognitive Tasks

##### Card rotations test

This is a pen-and-paper test of mental rotation with 20 target figures presented with eight other alternative illustrations of it on its right (Ekstrom et al., 1976). Among these eight alternative figures, some are rotated versions of the target figure while others are mirror images of the target figure. For each of these figures, the participant must decide whether it appears to be the same as (i.e., rotated in the picture plane) or different from (i.e., mirror image) the target figure. The test has two parts and the dependent measure was the total number of correct items minus the total number of incorrect items summed over these two parts. The maximum score attainable is 160.

##### Landmark recognition memory task

This task assessed the ability to recognize previously encountered landmarks in the virtual maze. It comprised of 48 trials showing the eight landmarks from the maze and eight “foil” landmarks that served as distractors. Each landmark was enlarged to the same scale and presented at the center of a gray background. Each landmark was presented three times in a randomized fashion leading to 48 trials. This repetition of landmark displays was made due to the fact that there were only four intersections in the maze, which were established in order to ensure that maze learning occurred at an appropriate level of difficulty for older adults. This practice of redisplaying pictures of landmarks or objects for recognition purpose has also been espoused by other researchers (e.g., Janzen, 2006). Consequently, 24 trials presented the landmarks that were found in the maze. On each trial, the participants were instructed that they must decide on whether they had seen the landmark in the maze by saying either ‘yes’ or ‘no.’ The experimenter tested every participant individually and recorded the verbal response on each trial. There was no time limit and the participants were instructed to respond as accurately as they could. The dependent measures recorded from each participant pertained respectively to the number of correctly recognized landmarks at decision and non-decision points in the maze and the number of correctly rejected foil landmarks. These recorded measures were converted to sensitivity index scores (*d’*) (Stanislaw and Todorov, 1999) that represented participants’ ability to discern between previously seen and distractor landmarks. They were computed for each participant by subtracting the proportion of foil landmarks that were incorrectly recognized (‘false alarms’) from the proportion of correctly recognized landmarks in the maze (‘hits’). Due to the number of trials showing critical and non-critical landmarks respectively (12) being less than that showing foil landmarks (24), all of the percentage scores were converted to *z*-scores to ensure standardized comparisons. For the two sets of *d*’ scores reflecting the corrected hits of critical and non-critical landmarks were computed based on: *d’* = *Z* (hits) – *Z* (false alarms) (see Macmillan and Creelman, 2005).

##### Landmark-direction association task

This task comprised 20 trials that required making decisions about the direction of travel in the virtual maze. A first-person still-photo of a particular location with a landmark appeared on each trial and the participant had to make a decision about the correct direction to take by pressing one of the arrow keys on the keyboard. Participants were asked “If you found yourself in this place in the maze, which direction would you travel?” On 12 trials, views of intersections were presented together with three directional arrows and the subject responded by indicating (i) forward, left, or right; (ii) backward, left, or right; (iii) left, forward, or backward; (iv) right, forward, or backward depending on the view. The participants decided on the directions by pressing arrow keys on the keyboard that corresponded to the relevant arrows on screen. On the remaining eight trials, views of the unidirectional alleys were presented together with two directional arrows in each trial and participants made their decisions with regards to three combinations of directional arrows: (i) forward or backward; (ii) left or backward; (iii) right or backward. These eight views were organized around four distinct landmarks and regarded as non-critical decision points for making directional decisions. Altogether, these 20 views were presented to participants in a randomized trial sequence. No time limit was imposed and the participants were instructed to respond as accurately as they could on each trial. The experimenter tested every participant individually and recorded the response on each trial. The dependent measures recorded from each participant pertained to the number of correct directional decisions made at the critical and non-critical decision points respectively.

## Results

### Virtual Maze Learning

**FIGURE 3:**
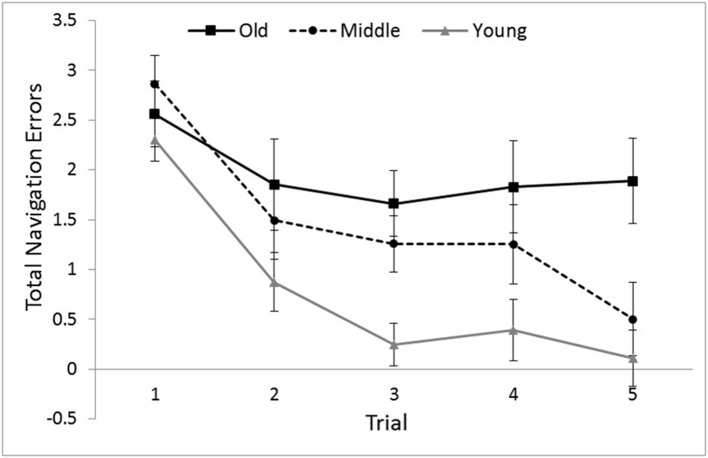
**Total number of navigation errors committed as a function of Age Group and Trial.** Adjusted means and *SE*s are shown after controlling for the covariate effect of computer experience. Younger adults committed fewer errors overall. Error bars indicate ± 1 *SE*.

To examine the potential gender and age group differences from the learning of the maze over the five trials, a mixed-model ANCOVA was performed with Age Group and Gender as the independent variables, navigation errors on each Trial as the within-subjects factor and computer experience as the covariate. There was a significant main effect of age, *F*(2,107) = 6.36, *p* = 0.002, η^2^ = 0.106. *Post hoc* Bonferroni pairwise comparisons showed that older adults committed more errors than younger adults (*p* = 0.002) and marginally more than middle-aged adults (*p* = 0.061). There was also a significant main effect of Trial, *F*(4,104) = 17.04, *p* < 0.001, η^2^ = 0.396 (see **Figure 3**) but no significant interaction between Trial and Age Group, *F*(8,210) = 1.16, *p* = 0.324, η^2^ = 0.042. *Post hoc* Bonferroni pairwise comparisons showed that the number of errors committed on the first trial was significantly greater than that committed on any subsequent trial (*p* < 0.001). Moreover, there was a significant main effect of Gender, *F*(1,107) = 6.59, *p* = 0.012, η^2^ = 0.058, with female adults committing more navigation errors (*M* = 1.72*, SD* = 1.31) than males (*M* = 1.09, *SD* = 1.30). Gender interacted significantly with Age Group, *F*(1,107) = 4.08, *p* = 0.020, η^2^ = 0.078 and with both Age Group and Trial, *F*(8,210) = 2.82, *p* = 0.006, η^2^ = 0.097.

**FIGURE 4:**
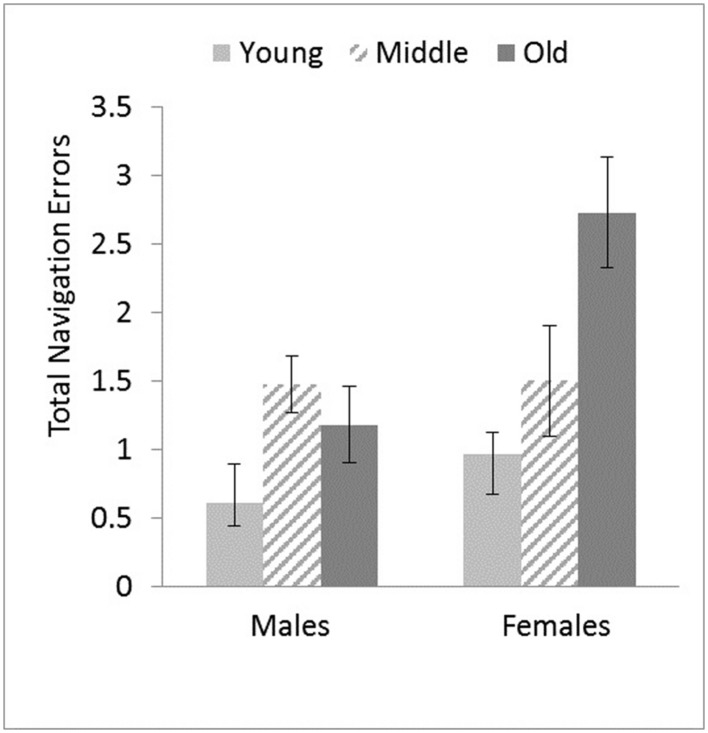
**Total number of navigation errors committed as a function of Age Group and Gender.** Adjusted means and *SE*s are shown after controlling for the covariate effect of computer experience. Older female adults committed the highest number of errors overall. Error bars indicate ± 1 *SE*.

The Gender × Age Group interaction was attributed to female adults committing more errors than male adults in both younger, *F*(1,55) = 6.89, *p* = 0.011, η^2^ = 0.111 and older age groups, *F*(1,24) = 4.51, *p* = 0.044, η^2^ = 0.158, (see **Figure 4**). *Post hoc* Bonferroni comparisons showed that younger females committed significantly fewer errors than both middle-aged (*p* = 0.05) and older females (*p* = 0.003) whereas younger males committed significantly fewer errors than middle-aged males only (*p* = 0.010).

**FIGURE 5:**
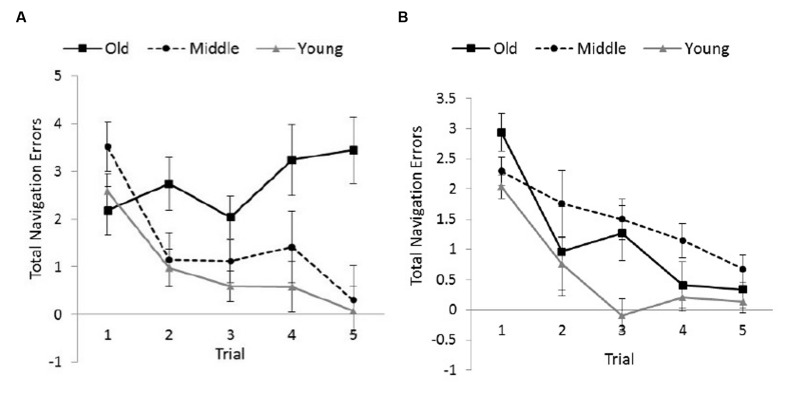
**Total number of navigation errors committed by female (A) and male participants (B) as a function of Age Group and Trial.** Adjusted means and *SE*s are shown after controlling for the covariate effect of computer experience. Compared to other groups, older females showed little improvement across trials. Error bars indicate ± 1 *SE*.

As for the three-way interaction of Gender with Age Group and Trial, it resulted from the Trial × Age Group interaction being significant among the female adults, *F*(8,108) = 2.19, *p* = 0.034, η^2^ = 0.139 (see **Figure 5A**), but non-significant among the male adults, *F*(8,96) = 1.33, *p* = 0.238, η^2^ = 0.100 (see **Figure 5B**). Older females exhibited a learning slope that was relatively flat across the trials (*M* = 2.73, *SD* = 0.63), with no pairs of trials differing significantly in navigation errors based on an alpha of 0.01 (Bonferroni corrected). This slope was in direct contrast to the learning slopes of the other groups of females and male adults, all of which exhibited a distinctive downward trend across the trials.

### Memory for Landmarks and Landmark-Direction Associations

As the initial maze learning trials were self-directed and self-paced, there were individual differences in the frequency with which each participant encountered a landmark. That is, people who made more errors and spent more time in the maze would see the landmarks more frequently and from a variety of different viewpoints. In the analyses of the performance on the tests of landmark recognition memory and landmark-direction association that followed maze learning, an additional measure of the total number of viewing events was computed. As the age groups were found to differ on this measure, *F*(2,111) = 16.23, *p* < 0.001, η^2^ = 0.226, with older adults having significantly more landmark views (*M* = 15.44, *SD* = 6.03) than both middle-aged (*M* = 11.31, 6.03) (*p* = 0.031) and younger adults (*M* = 7.55, *SD* = 6.03) (*p*< 0.001) s, this measure was added to the univariate analyses as a covariate to ensure that age differences on the cognitive task measures were not confounded by age differences in the extent of visual exposure to the different cues.

#### Landmark Recognition Memory

**FIGURE 6:**
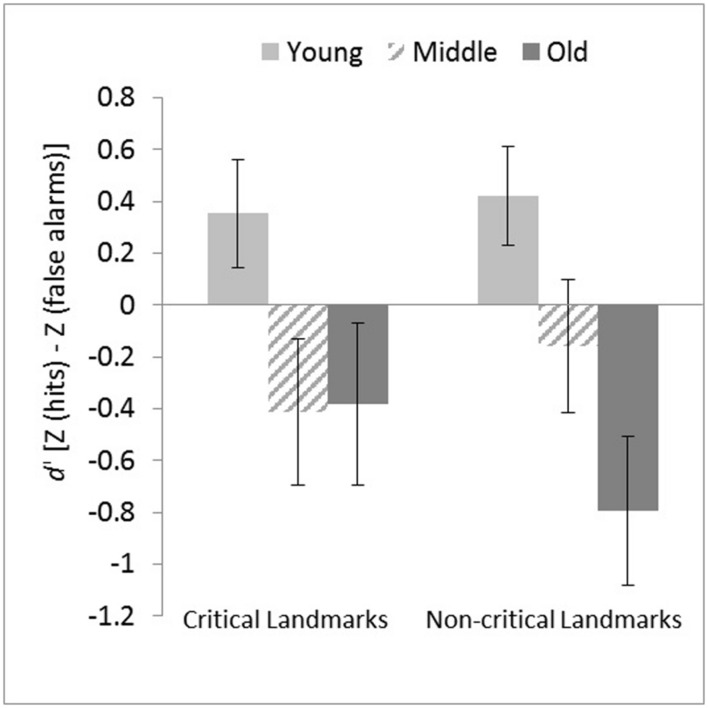
***d*’ scores on the Landmark Recognition Memory Task from all participants as a function of age group and landmark cue type.** Adjusted means and *SE*s are shown after controlling for the covariate effect of total viewing events. Error bars indicate ± 1 *SE*.

To analyze the performance on the landmark recognition memory test, a mixed-model ANCOVA was performed with Age Group and Gender as the independent variables, *d’* scores from the correct recognition of critical and non-critical landmark cues (located at decision and non-decision points respectively) as the repeated dependent measure and total viewing events as the covariate. There was a significant main effect of age, *F*(2,107) = 4.43, *p* = 0.014, η^2^ = 0.045, with younger adults attaining higher *d’* scores than both middle-aged and older adults. There was no significance difference between the two sets of landmark recognition *d’* scores, *F*(1,107) = 1.99 × 10^-4^, *p* = 0.989, η^2^ < 0.001. The interaction between Age Group and Landmark Cue Type was significant, *F*(2,107) = 3.15, *p* = 0.047, η^2^ = 0.056. This interaction was due to the age groups differing significantly in non-critical landmark recognition, *F*(2,107) = 5.75, *p* = 0.004, η^2^ = 0.095, but not in critical landmark recognition, *F*(2,107) = 2.92, *p* = 0.058, η^2^ = 0.050 (see **Figure 6**). *Post hoc* Bonferroni comparisons showed that younger adults attained significantly higher *d’* scores than older adults (*p* = 0.003) in the recognition of non-critical landmark cues. Gender did not produce any significant main effect and did not interact significantly with Age Group and/or Landmark Cue Type, *F*s < 2.09, *p*s > 0.12.

#### Landmark-Direction Associative Learning

To analyze the performance on the landmark-direction association task, a mixed-model ANCOVA was performed with Age Group and Gender as the independent variables, percentage accuracy scores from the two types of landmark cues (shown at decision and non-decision points respectively) as the repeated dependent measure and total viewing events as the covariate. In view of the difference in chance-level performance between critical (chance = 33.33%) and non-critical landmark scenes (chance = 50%) that elicited a discrepancy in mean performance values, we converted all the percentage accuracy scores from each landmark condition to *z*-scores to render a more precise portrayal of the pattern of within-subjects differences across the age groups.

The results showed a significant main effect of age, *F*(2,107) = 7.54, *p* < 0.001, η^2^ = 0.123, after controlling for the significant covariate effect of total viewing events, *F*(1,107) = 5.44, *p* = 0.022, η^2^ = 0.048. *Post hoc* Bonferroni pairwise comparisons showed that younger adults attained significantly higher percentage accuracy scores (*M* = 76.29, *SD* = 15.31; *Z* = 0.30) than both middle-aged (*M* = 71.14, *SD* = 14.49; *Z* = 0.04) (*p* = 0.047) and older adults (*M* = 61.32, *SD* = 15.69; *Z* = -0.48) (*p* = 0.001). There was no significant interaction between Age Group and Landmark Cue Type, *F*(2,107) = 2.35, *p* = 1.00, η^2^ = 0.042. Gender did not produce any significant main effect and did not interact significantly with Age Group and/or Landmark Cue Type, *F*s < 1.0, *p*s > 0.66.

#### Card Rotation

An ANOVA with Age Group and Gender as the independent variables and card rotation accuracy as the dependent variable showed a significant main effect of age, *F*(2,108) = 7.27, *p* = 0.001, η^2^ = 0.119. *Post hoc* comparisons using Tukey *HSD* showed that younger adults attained significantly higher scores (*M* = 104.02, *SD* = 30.64) than older adults (*M* = 75.04, *SD* = 29.61) (*p* < 0.001). Middle-aged adults’ scores (*M* = 88.17, *SD* = 32.49) did not differ significantly from either group (*p*s > 0.05). There was also a significant main effect of gender, *F*(1,108) = 7.40, *p* = 0.008, η^2^ = 0.064, with males scoring higher (*M* = 102.75, *SD* = 32.24) than females (*M* = 84.68, *SD* = 31.17). The interaction between Age Group and Gender was not significant, *F*(2,108) = 2.16, *p* = 0.12, η^2^ = 0.038.

#### Correlational Analyses

We tested the relationships between navigation errors and landmark-direction association accuracy from each navigation point (in *z*-scores), landmark recognition discriminability of each cue type and card-rotation accuracy. For navigation errors, we used the sum of all errors over the five trials. As shown in **Table 2**, there were some common findings across the age groups: cumulative navigation errors were moderately and negatively correlated with the landmark-direction accuracy scores from decision points in each of the three groups (-0.35 ≤*r* ≤-0.27, *p*s < 0.10), as well as with the card rotation accuracy scores of younger [*r*(58) = -0.32, *p* = 0.015] and older adults [*r*(27) = -0.65, *p* < 0.001]. However, older adults were exceptional for having positive and moderate correlations between cumulative navigation errors and recognition *d’* scores for both critical [*r*(27) = 0.39, *p* = 0.045] and non-critical landmarks [*r*(27) = 0.42, *p* = 0.031].

**Table 2:** Pearson product-moment correlations between cumulative navigation errors and cognitive task measures in each age group.

	Cumulative navigation errors		
	Younger adults	Middle-aged adults	Older adults
Critical landmarks *d’*	-0.02	-0.18	0.39ˆ*
Non-critical landmarks *d’*	-0.05	-0.29	0.42ˆ*
Landmark-direction association ACC (Decision Points)	-0.27ˆ*	-0.34ˆ†	-0.35ˆ†
Landmark-direction association ACC (Non-decision Points)	-0.17	-0.08	-0.09
Card otation ACC	-0.32ˆ**	-0.08	-0.65ˆ**

*^†^p < 0.10; ^∗^p< 0.05; ^∗∗^p < 0.01.*

## Discussion

This study examined the differences between younger, middle-aged and older adults with regards to their navigation performance in a virtual maze and subsequent performance in landmark recognition memory, landmark-direction associative learning and visuospatial ability. Overall, younger adults outperformed older adults in all the tasks except in critical landmark recognition. They also outperformed middle-aged adults in the recall of heading directions from decision and non-decision points and in card rotation performance. Middle-aged adults outperformed older adults on all the task measures but the differences did not reach significance. Correlations between the task measures further showed different patterns of relationships between the navigation performance and cognitive task performance measures across the age groups.

Closer examinations starting with total navigation errors showed that older adults committed significantly more errors than younger adults. These findings were consistent with previous studies that showed an age-related differences in the efficiency of route learning, particularly with regards to the planning or selection of the best route to reach a particular destination (e.g., Moffat et al., 2001; Allain et al., 2005; Salthouse and Siedlecki, 2007). Likewise, the prominent differences between younger and older adults in terms of landmark-direction association replicated previous findings which showed older adults to be relatively poorer at such associative learning (Head and Isom, 2010). Moreover, older adults’ lower level of mental rotation ability (as represented by their card rotation accuracy scores) was consistent with those found in previous studies (e.g., Berg et al., 1982; Hertzog and Rypma, 1991). Card rotation accuracy scores were negatively correlated with cumulative navigation errors among the older adults (i.e., lower rotation accuracy, more navigation errors), suggesting that older adults’ poorer route learning could be partly accounted by poorer mental rotation ability or spatial working memory capacity than younger adults (Hertzog and Rypma, 1991).

Younger adults’ demonstration of better memory for non-critical landmarks was contrary to our prediction that older adults would recognize relatively more non-critical landmarks. It seems that older adults’ predisposition toward encoding distractors (Bell et al., 2008; Campbell et al., 2010; Amer and Hasher, 2014) did not similarly apply to the encoding of irrelevant landmark cues in the navigational context of a virtual maze. However, it must be acknowledged that even though we deliberately placed irrelevant cues in the virtual maze, this environment was simpler and contained fewer cues than would be encountered in the real-world. Navigating an actual city, for example, would present a plethora of navigationally irrelevant cues that may prove to be much more distracting.

In addition, a somewhat paradoxical finding was that item recognition memory for critical and non-critical landmarks was *positively* correlated with navigation errors (i.e., better recognition memory, poorer navigation performance) in the older adults. These findings suggest that older adults who attend to and encode item/object characteristics (leading to better item recognition memory) may do this at the expense of linking the objects to directions, culminating in poorer landmark-direction associative learning and poorer navigation performance.

It is also noteworthy that both middle-aged and older adults seem to experience a common deficit in associative memory for landmark-direction associations. This pattern of results is analogous to those from previous studies in which participants were instructed to utilize a particular memory strategy for binding together paired associates (e.g., for word pairs, see Naveh-Benjamin, 2000; Naveh-Benjamin et al., 2007; for name-face pairs, see Naveh-Benjamin et al., 2009). Owing to the fact that this study employed incidental learning with no instructions about strategy, this suggests that middle-aged and older adults might have adopted self-initialized strategies that led them to focus more on the object characteristics of the landmarks than on the relations of those landmarks with spatial or contextual information (i.e., heading directions, background scenes). Consequently, this might lead both age groups to have fewer cognitive resources or lower cognitive capacity than younger adults for the formation of appropriate landmark-direction associations. This interpretation is supported by previous research which showed that older adults have reduced cognitive resources for episodic or associative memory formation under conditions where they need to allocate more attentional resources to the processing of individual items instead of the relations between them (e.g., Craik and Byrd, 1982; Craik, 1983; Naveh-Benjamin et al., 2005; Naveh-Benjamin and Kilb, 2014).

Furthermore, the relatively high performance of younger adults in the landmark-direction association task could reflect their more effective use of spatial representation strategies. Such strategies generally pertain to an orientation strategy that focuses on the estimation and tracking of one’s orientation relative to surrounding landmarks (Lawton, 1994, 1996; Pazzaglia and De Beni, 2001; Lawton and Kallai, 2002; Kato and Takeuchi, 2003) and an allocentric strategy that focuses on visualizing interobject relations and forming a cognitive map from an environment-centered perspective (Pazzaglia and De Beni, 2001; Hegarty et al., 2002; Rodgers et al., 2012). As the landmark-direction association task involved selecting directions based on viewpoints that were not necessarily directly encountered, a potentially more effective use of a spatial orientation strategy by younger adults might have facilitated their learning of landmark-direction pairings from multiple viewpoints, eventually enabling them to outperform both middle-aged and older adults on the task. Consistent with evidence showing that younger adults were stronger adherents of the allocentric strategy than older adults when navigating a two-choice Y-maze (Rodgers et al., 2012), younger adults most probably conducted better cognitive mapping of the spatial relations between the landmarks, leading them to commit substantially fewer navigation errors than older adults.

Another novel aspect of this study pertains to the gender effects that affected navigation performance. Older female adults—unlike the other female adults and male adults, all of whom demonstrated learning across repeated trials—were exceptional for not showing any noticeable decline in navigation errors with more trial exposure. This led to them committing substantially greater navigation errors than older males—a difference that might be attributed to gender differences in navigation strategy use (i.e., males prefer to use metric-based/spatial information whereas females prefer to use landmark information) (e.g., see Lawton, 1994, 1996; Dabbs et al., 1998; Lawton and Kallai, 2002; Saucier et al., 2002). Specifically, older females’ potential use of a non-spatial landmark strategy that focuses on processing landmark/object characteristics (Dabbs et al., 1998; Saucier et al., 2002) might have deterred them from learning the spatial layout of the virtual maze, thereby leading them to maintain a relatively high level of navigation errors across the trials. Further research on relating potential gender differences in navigation strategy use and navigational performance among older adults is needed to clarify this possibility.

Along with the implications of the behavioral findings above, it is worth noting that age-differences in spatial navigation and associative learning may be underpinned by age-related differences in brain-related processes. Extant fMRI studies have demonstrated that activation in the hippocampal/parahippocampal region among non-demented older adults, when compared to younger adults, was either reduced (Meulenbroek et al., 2004; Moffat et al., 2006) or absent (Antonova et al., 2009) during the performance of navigational tasks. Moreover, activation in the parahippocampal gyrus has been related to encoding of salient and navigationally relevant landmarks and their corresponding positions in space (Aguirre et al., 1996; Maguire et al., 1998; Janzen et al., 2007). For instance, when navigating a maze-like virtual museum, the parahippocampal gyrus was shown to have markedly higher activation in response to landmarks located at decision points compared to those located at non-decision points (Janzen and van Turennout, 2004; Janzen and Weststeijn, 2007; Janzen et al., 2007; Wegman and Janzen, 2011). These fMRI studies were complemented by structural MRI studies which further highlighted the hippocampus as one of the brain areas that exhibit shrinkage in volume with increased age (Raz et al., 2005) and a positive relationship between its volume and navigational performance (Moffat et al., 2007; Head and Isom, 2010; Schinazi et al., 2013). Cumulatively, these studies suggest that age-related changes in the functional and structural properties of the hippocampal/parahippocampal formation may have led to the age-related differences in navigational performance observed in our virtual maze task.

Likewise, in consideration of associative learning and memory, there have been studies that implicated the hippocampus as pivotal for the automatic binding of information (e.g., Eichenbaum and Bunsey, 1995; Henke et al., 1997; Wallenstein et al., 1998). These automatic binding processes, normally activated under incidental learning conditions, have also been shown to be negatively affected in older adults (e.g., see Moscovitch, 1994; Grady et al., 1995; Mitchell et al., 2000). In tasks that require the binding or combined encoding of different features, older adults have been shown to have lower activation in the hippocampus and prefrontal cortex than younger adults (e.g., Mitchell et al., 2000; Dennis et al., 2008). The prefrontal and hippocampal regions have also been proposed to be involved in strategic-effortful and automatic binding processes respectively (Moscovitch and Winocur, 1992; Moscovitch, 1994) and subsequent neurocomputational research has shown that senescent changes in the neuromodulatory mechanisms underlying the fronto-hippocampal circuitry may play a basic role in accounting for the associative deficit of older adults (e.g., see Li et al., 2001, 2005; Li and Sikström, 2002). Taken together, these developments makes it relevant for future studies to investigate the associative learning of landmark and directional information at the neural and systems levels, particularly with regards to the functional connectivity between the prefrontal cortex and the hippocampus.

Finally, by understanding the specific cognitive and neural factors that affect navigational decline in the normal aging population and comparing them to corresponding factors affecting patients with mild cognitive impairment and Alzheimer’s disease (AD), we hope that a better identification of reliable markers of AD onset will emerge (Lithfous et al., 2013). Such an understanding will also be beneficial to the development of spatial navigation training (Lövdén et al., 2012) and/or pedestrian navigation aid devices (e.g., see Goodman et al., 2005) that cater to the special needs of older adults with different levels of navigational ability.

## Author Contributions

SM conceived and designed the experiment. JZ analyzed and the data and drafted the manuscript. JZ and SM revised the manuscript to ensure accuracy of all sections. Both authors approved the current final version for publication.

## Conflict of Interest Statement

The authors declare that the research was conducted in the absence of any commercial or financial relationships that could be construed as a potential conflict of interest.

## References

[B1] Adamo D. E. Briceno E. M. Sindone J. A. Alexander N. B. Moffat S. D. (2012). Age differences in virtual environment and real world path integration. *Front. Aging Neurosci.* 4:26 10.3389/fnagi.2012.00026 PMC3457005 23055969

[B2] Aguirre G. K. Detre J. A. Alsop D. C. D’Esposito M. (1996). The parahippocampus subserves topographical learning in 4man. *Cereb. Cortex* 6 823–829. 10.1093/cercor/6.6.823 8922339

[B3] Allain P. Nicoleau S. Pinon K. Etcharry-Bouyx F. Barre J. Berrut G. (2005). Executive functioning in normal aging: a study of action planning using the Zoo Map Test. *Brain Cogn.* 57 4–7. 10.1016/j.bandc.2004.08.011 15629206

[B4] Amer T. Hasher L. (2014). Conceptual processing of distractors by older but not younger adults. *Psychol. Sci.* 25 2252–2258. 10.1177/0956797614555725 25376192

[B5] Antonova E. Parslow D. Brammer M. Dawson G. R. Jackson S. H. Morris R. G. (2009). Age-related neural activity during allocentric spatial memory. *Memory* 17 125–143. 10.1080/09658210802077348 18608980

[B6] Bell R. Buchner A. Mund I. (2008). Age-related differences in irrelevant-speech effects. *Psychol. Aging* 23 377–391. 10.1037/0882-7974.23.2.377 18573011

[B7] Berg C. Hertzog C. Hunt E. (1982). Age differences in the speed of mental rotation. *Dev. Psychol.* 18 95–107. 10.1037/0012-1649.18.1.95

[B8] Blades M. Medlicott L. (1992). Developmental Differences in the ability to give route directions from a map. *J. Environ. Psychol.* 12 175–185. 10.1016/S0272-4944(05)80069-6

[B9] Campbell K. L. Hasher L. Thomas R. C. (2010). Hyper-binding: a unique age effect. *Psychol. Sci.* 21 399–405. 10.1177/0956797609359910 20424077 PMC3399902

[B10] Cohen R. Schuepfer T. (1980). The representation of landmarks and routes. *Child Dev.* 51 1065–1071. 10.2307/1129545

[B11] Connelly S. L. Hasher L. Zacks R. T. (1991). Age and Reading: the impact of distraction. *Psychol. Aging* 6 533–541. 10.1037/0882-7974.6.4.533 1777141

[B12] Craik F. I. M. (1983). On the transfer of information from temporary to permanent memory. *Philos. Trans. R. Soc. B* 302 341–359. 10.1098/rstb.1983.0059

[B13] Craik F. I. M. Byrd M. (1982). “Aging and cognitive deficits: the role of attentional resources,” in *Advances in the Study of Communication and Affect: Aging and Cognitive Processes* Vol. 8 eds Craik F. I. M. Trehub S. E. (New York, NY: Plenum Press), 191–211.

[B14] Dabbs J. M. Chang E. L. Strong R. A. Milun R. (1998). Spatial ability, navigation strategy, and geographic knowledge among men and women. *Evol. Hum. Behav.* 19 89–98. 10.1016/S1090-5138(97)00107-4

[B15] Daniel M. P. Denis M. (1998). Spatial descriptions as navigational aids: a cognitive analysis of route directions. *Kognitionswissenschaft* 7 45–52. 10.1007/BF03354963

[B16] Dennis N. A. Hayes S. M. Prince S. E. Madden D. J. Huettel S. A. Cabeza R. (2008). Effects of aging on the neural correlates of successful item and source memory encoding. *J. Exp. Psychol. Learn. Mem. Cogn.* 34 791–808. 10.1037/0278-7393.34.4.791 18605869 PMC2752883

[B17] Driscoll I. Hamilton D. A. Petropoulos H. Yeo R. A. Brooks W. M. Baumgartner R. N. (2003). The aging hippocampus: cognitive, biochemical and structural findings. *Cereb. Cortex* 13 1344–1351. 10.1093/cercor/bhg081 14615299

[B18] Duchek J. M. Balota D. A. Thessing V. C. (1998). Inhibition of visual and conceptual information during reading in healthy aging and Alzheimer’s disease. *Neuropsychol. Dev. Cogn. B Aging Neuropsychol. Cogn.* 5 169–181. 10.1076/anec.5.3.169.616 25233057

[B19] Eichenbaum H. Bunsey M. (1995). On the binding of associations in memory: clues from studies on the role of the hippocampal region in paired-associate learning. *Curr. Dir. Psychol. Sci.* 4 19–23. 10.1111/1467-8721.ep10770954

[B20] Ekstrom R. French J. Harman H. (1976). *Manual for Kit of Factor Referenced Cognitive Tests.* Princeton, NJ: Educational Testing Service.

[B21] Folstein M. F. Folstein S. E. McHugh P. R. (1975). Mini-mental state: a practical method for grading the cognitive state of patients for the clinician. *J. Psychiatr. Res.* 12 189–198. 10.1016/0022-3956(75)90026-6 1202204

[B22] Goodman J. Brewster S. A. Gray P. (2005). How can we best use landmarks to support older people in navigation? *Behav. Inf. Technol.* 24 3–20. 10.1080/01449290512331319021

[B23] Grady C. L. Mcintosh A. R. Horwitz B. Maisog J. M. Ungerleider L. G. Mentis M. J. (1995). Age-related reductions in human recognition memory due to impaired encoding. *Science* 269 218–221. 10.1126/science.7618082 7618082

[B24] Harris M. A. Wolbers T. (2012). Ageing effects on path integration and landmark navigation. *Hippocampus* 22 1770–1780. 10.1002/hipo.22011 22431367

[B25] Harris M. A. Wolbers T. (2014). How age-related strategy switching deficits affect wayfinding in complex environments. *Neurobiol. Aging* 35 1095–1102. 10.1016/j.neurobiolaging.2013.10.086 24239438

[B26] Head D. Isom M. (2010). Age effects on wayfinding and route learning skills. *Behav. Brain Res.* 209 49–58. 10.1016/j.bbr.2010.01.012 20085784

[B27] Hegarty M. Richardson A. E. Montello D. R. Lovelace K. Subbiah I. (2002). Development of a self-report measure of environmental spatial ability. *Intelligence* 30 425–447. 10.1016/S0160-2896(02)00116-2

[B28] Henke K. Buck A. Weber B. Wieser H. G. (1997). Human hippocampus establishes associations in memory. *Hippocampus* 7 249–256. 10.1002/(SICI)1098-10631997)7:3<249::AID-HIPO1>3.0.CO;2-G 9228523

[B29] Hertzog C. Rypma B. (1991). Age-differences in components of mental-rotation task-performance. *Bull. Psychon. Soc.* 29 209–212. 10.3758/BF03335237

[B30] Iaria G. Palermo L. Committeri G. Barton J. J. S. (2009). Age differences in the formation and use of cognitive maps. *Behav. Brain Res.* 196 187–191. 10.1016/j.bbr.2008.08.040 18817815

[B31] Jansen P. Schmelter A. Heil M. (2010). Spatial knowledge acquisition in younger and elderly adults: a study in a virtual environment. *Exp. Psychol.* 57 54–60. 10.1027/1618-3169/a000007 20178963

[B32] Jansen-Osmann P. (2002). Using desktop virtual environments to investigate the role of landmarks. *Comput. Hum. Behav.* 18 427–436. 10.1016/S0747-5632(01)00055-3

[B33] Jansen-Osmann P. Wiedenbauer G. (2004). The representation of landmarks and routes in children and adults: a study in a virtual environment. *J. Environ. Psychol.* 24 347–357. 10.1016/j.jenvp.2004.08.003

[B34] Janzen G. (2006). Memory for object location and route direction in virtual large-scale space. *Q. J. Exp. Psychol.* 59 493–508. 10.1080/02724980443000746 16627352

[B35] Janzen G. van Turennout M. (2004). Selective neural representation of objects relevant for navigation. *Nat. Neurosci.* 7 673–677. 10.1038/nn1257 15146191

[B36] Janzen G. Wagensveld B. van Turennout M. (2007). Neural representation of navigational relevance is rapidly induced and long lasting. *Cereb. Cortex* 17 975–981. 10.1093/cercor/bhl008 16751297

[B37] Janzen G. Weststeijn C. G. (2007). Neural representation of object location and route direction: an event-related fMRI study. *Brain Res.* 1165 116–125. 10.1016/j.brainres.2007.05.074 17651709

[B38] Kato Y. Takeuchi Y. (2003). Individual differences in wayfinding strategies. *J. Environ. Psychol.* 23 171–188. 10.1016/S0272-4944(03)00011-2

[B39] Kirasic K. C. (1991). Spatial cognition and behavior in young and elderly adults: implications for learning new environments. *Psychol. Aging* 6 10–18. 10.1037/0882-7974.6.1.10 2029357

[B40] Lawton C. A. (1994). Gender differences in way-finding strategies: relationship to spatial ability and spatial anxiety. *Sex Roles* 30 765–779. 10.1007/Bf01544230

[B41] Lawton C. A. (1996). Strategies for indoor wayfinding: the role of orientation. *J. Environ. Psychol.* 16 137–145. 10.1006/jevp.1996.0011

[B42] Lawton C. A. Kallai J. (2002). Gender differences in wayfinding strategies and anxiety about wayfinding: a cross-cultural comparison. *Sex Roles* 47 389–401. 10.1023/A:1021668724970

[B43] Li S. C. Lindenberger U. Sikstrom S. (2001). Aging cognition: from neuromodulation to representation. *Trends Cogn. Sci.* 5 479–486. 10.1016/S1364-6613(00)01769-1 11684480

[B44] Li S. C. Naveh-Benjamin M. Lindenberger U. (2005). Aging neuromodulation impairs associative binding: a neurocomputational account. *Psychol. Sci.* 16 445–450. 10.1111/j.0956-7976.2005.01555.x 15943670

[B45] Li S. C. Sikström S. (2002). Integrative neurocomputational perspectives on cognitive aging, neuromodulation, and representation. *Neurosci. Biobehav. Rev.* 26 795–808. 10.1016/S0149-7634(02)00066-0 12470691

[B46] Lipman P. D. (1991). Age and exposure differences in acquisition of route information. *Psychol. Aging* 6 128–133. 10.1037/0882-7974.6.1.128 2029361

[B47] Lithfous S. Dufour A. Despres O. (2013). Spatial navigation in normal aging and the prodromal stage of Alzheimer’s disease: insights from imaging and behavioral studies. *Ageing Res. Rev.* 12 201–213. 10.1016/j.arr.2012.04.007 22771718

[B48] Lövdén M. Schaefer S. Noack H. Bodammer N. C. Kuhn S. Heinze H. J. (2012). Spatial navigation training protects the hippocampus against age-related changes during early and late adulthood. *Neurobiol. Aging* 33 620.e9–620.e22. 10.1016/j.neurobiolaging.2011.02.013 21497950

[B49] Macmillan N. A. Creelman D. (2005). *Detection Theory: A User’s Guide*, 2nd Edn. Mahwah, NJ: Erlbaum.

[B50] Maguire E. A. Frith C. D. Burgess N. Donnett J. G. O’Keefe J. (1998). Knowing where things are: parahippocampal involvement in encoding object locations in virtual large-scale space. *J. Cogn. Neurosci.* 10 61–76. 10.1162/089892998563789 9526083

[B51] Mahmood O. Adamo D. Briceno E. Moffat S. D. (2009). Age differences in visual path integration. *Behav. Brain Res.* 205 88–95. 10.1016/j.bbr.2009.08.001 19665496

[B52] May C. P. (1999). Synchrony effects in cognition: the costs and a benefit. *Psychon. Bull. Rev.* 6 142–147. 10.3758/Bf03210822 12199309

[B53] McGinnis D. (2012). Susceptibility to distraction during reading in young, young-old, and old-old adults. *Exp. Aging Res.* 38 370–393. 10.1080/0361073x.2012.699365 22830665

[B54] Meulenbroek O. Petersson K. M. Voermans N. Weber B. Fernandez G. (2004). Age differences in neural correlates of route encoding and route recognition. *Neuroimage* 22 1503–1514. 10.1016/j.neuroimage.2004.04.007 15275907

[B55] Mitchell K. J. Johnson M. K. Raye C. L. D’Esposito M. (2000). fMRI evidence of age-related hippocampal dysfunction in feature binding in working memory. *Cogn. Brain Res.* 10 197–206. 10.1016/S0926-6410(00)00029-X 10978709

[B56] Moffat S. D. Elkins W. Resnick S. M. (2006). Age differences in the neural systems supporting human allocentric spatial navigation. *Neurobiol. Aging* 27 965–972. 10.1016/j.neurobiolaging.2005.05.011 15982787

[B57] Moffat S. D. Kennedy K. M. Rodrigue K. M. Raz N. (2007). Extrahippocampal contributions to age differences in human spatial navigation. *Cereb. Cortex* 17 1274–1282. 10.1093/cercor/bhl036 16857855

[B58] Moffat S. D. Resnick S. M. (2002). Effects of age on virtual environment place navigation and allocentric cognitive mapping. *Behav. Neurosci.* 116 851–859. 10.1037/0735-7044.116.5.851 12369805

[B59] Moffat S. D. Zonderman A. B. Resnick S. M. (2001). Age differences in spatial memory in a virtual environment navigation task. *Neurobiol. Aging* 22 787–796. 10.1016/S0197-4580(01)00251-2 11705638

[B60] Moscovitch M. (1994). Cognitive resources and dual-task interference effects at retrieval in normal people: the role of the frontal lobes and medial temporal cortex. *Neuropsychology* 8 524–534. 10.1037/0894-4105.8.4.524

[B61] Moscovitch M. Winocur G. (1992). “The neuropsychology of memory and aging,” in *The Handbook of Aging and Cognition*, eds Craik F. I. M. Salthouse T. A. (Hillsdale, NJ: Erlbaum), 315–372.

[B62] Naveh-Benjamin M. (2000). Adult age differences in memory performance: tests of an associative deficit hypothesis. *J. Exp. Psychol. Learn. Mem. Cogn.* 26 1170–1187. 10.1037/0278-7393.26.2.1170 11009251

[B63] Naveh-Benjamin M. Brav T. K. Levy O. (2007). The associative memory deficit of older adults: the role of strategy utilization. *Psychol. Aging* 22 202–208. 10.1037/0882-7974.22.1.202 17385995

[B64] Naveh-Benjamin M. Craik F. I. M. Guez J. Kreuger S. (2005). Divided attention in younger and older adults: effects of strategy and relatedness on memory performance and secondary task costs. *J. Exp. Psychol. Learn. Mem. Cogn.* 31 520–537. 10.1037/0278-7393.31.3.520 15910135

[B65] Naveh-Benjamin M. Kilb A. (2014). Age-related differences in associative memory: the role of sensory decline. *Psychol. Aging* 29 672–683. 10.1037/a0037138 25089854

[B66] Naveh-Benjamin M. Shing Y. L. Kilb A. Werkle-Bergner M. Lindenberger U. Li S. C. (2009). Adult age differences in memory for name-face associations: the effects of intentional and incidental learning. *Memory* 17 220–232. 10.1080/09658210802222183 18654927

[B67] Newman M. C. Kaszniak A. W. (2000). Spatial memory and aging: performance on a human analog of the Morris water maze. *Aging Neuropsychol. Cogn.* 7 86–93. 10.1076/1382-5585(200006)7:2;1-U;Ft086

[B68] O’Bryant S. E. Humphreys J. D. Smith G. E. Ivnik R. J. Graff-Radford N. R. Petersen R. C. (2008). Detecting dementia with the mini-mental state examination in highly educated individuals. *Arch. Neurol.* 65 963–967. 10.1001/archneur.65.7.963 18625866 PMC2587038

[B69] Old S. R. Naveh-Benjamin M. (2008). Differential effects of age on item and associative measures of memory: a meta-analysis. *Psychol. Aging* 23 104–118. 10.1037/0882-7974.23.1.104 18361660

[B70] Pazzaglia F. De Beni R. (2001). Strategies of processing spatial information in survey and landmark-centred individuals. *Eur. J. Cogn. Psychol.* 13 493–508. 10.1080/09541440042000124

[B71] Raz N. Lindenberger U. Rodrigue K. M. Kennedy K. M. Head D. Williamson A. (2005). Regional brain changes in aging healthy adults: general trends, individual differences and modifiers. *Cereb. Cortex* 15 1676–1689. 10.1093/cercor/bhi044 15703252

[B72] Rodgers M. K. Sindone J. A. Moffat S. D. (2012). Effects of age on navigation strategy. *Neurobiol. Aging* 33 202.e15–202.e22. 10.1016/j.neurobiolaging.2010.07.021 20832911 PMC4283776

[B73] Salthouse T. A. Siedlecki K. L. (2007). Efficiency of route selection as a function of adult age. *Brain Cogn.* 63 279–286. 10.1016/j.bandc.2006.09.006 17079064 PMC3838961

[B74] Saucier D. M. Green S. M. Leason J. MacFadden A. Bell S. Elias L. J. (2002). Are sex differences in navigation caused by sexually dimorphic strategies or by differences in the ability to use the strategies? *Behav. Neurosci.* 116 403–410. 10.1037/0735-7044.116.3.403 12049321

[B75] Schinazi V. R. Nardi D. Newcombe N. S. Shipley T. F. Epstein R. A. (2013). Hippocampal size predicts rapid learning of a cognitive map in humans. *Hippocampus* 23 515–528. 10.1002/hipo.22111 23505031 PMC3690629

[B76] Stanislaw H. Todorov N. (1999). Calculation of signal detection theory measures. *Behav. Res. Methods Instrum. Comput.* 31 137–149. 10.3758/Bf03207704 10495845

[B77] Wallenstein G. V. Eichenbaum H. Hasselmo M. E. (1998). The hippocampus as an associator of discontiguous events. *Trends Neurosci.* 21 317–323. 10.1016/S0166-2236(97)01220-4 9720595

[B78] Ward S. L. Newcombe N. Overton W. F. (1986). Turn left at the church, or 3 Miles north: a study of direction giving and sex differences. *Environ. Behav.* 18 192–213. 10.1177/0013916586182003

[B79] Wegman J. Janzen G. (2011). Neural encoding of objects relevant for navigation and resting state correlations with navigational ability. *J. Cogn. Neurosci.* 23 3841–3854. 10.1162/jocn_a_00081 21671733

[B80] Wiener J. M. de Condappa O. Harris M. A. Wolbers T. (2013). Maladaptive bias for extrahippocampal navigation strategies in aging humans. *J. Neurosci.* 33 6012–6017. 10.1523/JNEUROSCI.0717-12.2013 23554482 PMC6618910

[B81] Wilkniss S. M. Jones M. G. Korol D. L. Gold P. E. Manning C. A. (1997). Age-related differences in an ecologically based study of route learning. *Psychol. Aging* 12 372–375. 10.1037/0882-7974.12.2.372 9189997

